# Effect of modified Mediterranean diet supplemented with partial enteral nutrition in post-surgical patients with Crohn’s disease: a pilot clinical trial

**DOI:** 10.1017/S0007114526106588

**Published:** 2026-06-28

**Authors:** Xiaoxu Huang, Yan Chen, Zhifang Zhong, Shuyan Li, Jiakai Luo, Yang Tang, Wen Hu, Yunpiao Hou, Zexin Chen, Yunxian Zhou, Xiujun Liao, Qiao Yu, Pianhong Zhang

**Affiliations:** 1 Department of Nutrition, The Second Affiliated Hospital, Zhejiang University School of Medicine, Hangzhou, Zhejiang, People’s Republic of China; 2 Department of Gastroenterology, The Second Affiliated Hospital, Zhejiang University School of Medicine, Hangzhou, Zhejiang, People’s Republic of China; 3 Department of Nursing, The Second Affiliated Hospital, Zhejiang University School of Medicine, Hangzhou, Zhejiang, People’s Republic of China; 4 Department of Colorectal Surgery, The Second Affiliated Hospital, Zhejiang University School of Medicine, Hangzhou, Zhejiang, People’s Republic of China; 5 Department of Gastroenterology, The First Affiliated Hospital of Zhejiang Chinese Medical University (Zhejiang Provincial Hospital of Chinese Medicine), Hangzhou, Zhejiang, People’s Republic of China; 6 Department of Science and Research, The Second Affiliated Hospital, Zhejiang University School of Medicine, Hangzhou, Zhejiang, People’s Republic of China; 7 School of Nursing, Zhejiang Chinese Medical University, Hangzhou, Zhejiang, People’s Republic of China

**Keywords:** Crohn’s disease, Modified Mediterranean diet, Exclusive enteral nutrition, Pilot study

## Abstract

Dietary intervention represents a promising strategy for managing post-surgical patients with Crohn’s disease (CD). This study aims to evaluate the effects of a modified Mediterranean diet (MMD) supplemented with partial enteral nutrition (PEN) for 4–5 weeks on quality of life in post-surgical CD patients, compared with exclusive enteral nutrition (EEN). The study was conducted at The Second Affiliated Hospital of Zhejiang University School of Medicine. The primary outcome was quality of life, measured using the 22-item inflammatory bowel disease quality-of-life questionnaire (IBDQOL-22) at the end of the intervention. Secondary outcomes included nutritional status and disease-related characteristics. Among 115 screened patients, forty-six were randomised to either the EEN group (*n* 24) or the MMD supplemented with PEN group (*n* 22). Twenty-three patients in the EEN group and twenty-two in the MMD supplemented with PEN group completed the study and were included for analysis. At the end of the intervention, both groups achieved similar 22-item inflammatory bowel disease quality-of-life questionnaire (IBDQOL-22) scores (EEN *v*. MMD supplemented with PEN: 88·43 (sd 9·17) *v*. 87·57 (sd 7·38), *P* = 0·734). In addition, both groups exhibited comparable nutritional status and disease-related characteristics (all *P* > 0·05). These results suggest that MMD supplemented with PEN provides comparable clinical benefits to EEN in post-surgical CD patients and may serve as an alternative nutritional strategy.

## Graphical abstract

**Figure:**
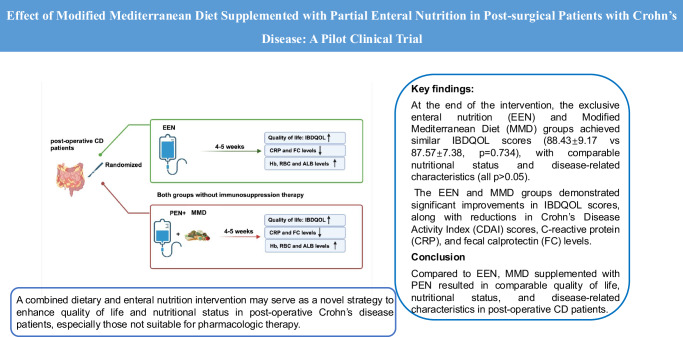

Crohn’s disease (CD) is a type of inflammatory bowel disease that can affect any part of the gastrointestinal tract, particularly the terminal ileum and proximal colon^(1)^. CD is characterised by a segmental distribution of lesions within the gastrointestinal tract, with chronic, progressive and recurrent transmural inflammation of the bowel wall that leads to wall thickening and luminal narrowing^(2)^. Current strategies for treating CD focus on achieving deep and long-term remission to improve quality of life while preventing complications and halting disease progression^(1)^. While anti-inflammation therapy is the first-line treatment for CD, approximately 80 % of CD patients still need to undergo surgery during their lifespan^(3)^, whereas 35 % needed a second or more operation^(4)^. Nearly one-third of post-surgical patients develop complications, highlighting the importance of post-surgical management^(4)^.

In post-surgical CD patients, dietary intervention offers a promising approach for disease management during the initial 4–6 weeks^(5–7)^. Importantly, dietary management is crucial for preventing post-surgical CD recurrence. Exclusive enteral nutrition (EEN), which involves using a complete, formulated liquid diet while excluding normal food components, can provide adequate nutrition and induce remission in up to 80 % of paediatric CD cases^(8,9)^. EEN also has minimal essential side effects and is linked to high rates of mucosal healing^(8)^. However, EEN is recommended only when drug therapy is declined or as an adjunct to standard treatments^(10)^. A major limitation of EEN for adults is poor adherence due to the unpleasant taste of the formulations, which often leads to treatment discontinuation and reduced efficacy. Moreover, by eliminating the social and hedonic aspects of food intake, EEN can negatively affect psychosocial well-being and overall quality of life.

Recent studies have introduced the Crohn’s Disease Exclusion Diet (CDED), a progressive whole-food diet that incorporates partial enteral nutrition (PEN). CDED is characterised by its high protein content and low levels of animal fat, heme, gluten and additives, while also promoting fibre intake^(11)^. This diet has demonstrated significant benefits for adult patients with CD in randomised trials^(11–13)^ and was initially recommended for managing mild-to-moderate active CD^(14)^. Additionally, the American Gastroenterological Association advises that all patients with inflammatory bowel disease, unless contraindicated, follow a Mediterranean diet. This diet emphasises fresh fruits, vegetables, monounsaturated fats, complex carbohydrates and lean proteins, while minimising consumption of ultra-processed foods, added sugars and salt to support overall health and well-being^(15)^. However, the taste and flavours of both the CDED and the Mediterranean diet have not been fully embraced by Chinese inflammatory bowel disease patients due to regional and dietary differences. Traditional Chinese eating habits are often based on rice or wheat as staple foods, complemented by a wide variety of vegetables, soy products, freshwater fish and light cooking methods^(16)^, whereas Mediterranean diets frequently incorporate olive oil, cheese and whole grains that may be less acceptable to Chinese patients. To address this challenge, we developed a Modified Mediterranean Diet (MMD) that aligns with the nutritional principles of the Mediterranean diet while incorporating culturally familiar Chinese ingredients and flavours.

Specifically, the MMD emphasises the use of rapeseed or soybean oil as primary sources of monounsaturated fats in place of olive oil and introduces soy products, tofu and freshwater fish as lean protein sources alongside moderate amounts of poultry. Whole grains are adapted to include brown rice, millet and oats, which are more acceptable in Chinese cuisine. The diet also integrates abundant seasonal vegetables and fruits, while maintaining reduced levels of red and processed meats, animal fats and refined sugars. In terms of culinary practices, the MMD encourages steaming, boiling and stir-frying with minimal oil to ensure palatability for Chinese patients.

This culturally adapted approach is designed to preserve the anti-inflammatory and metabolic benefits of the Mediterranean dietary pattern while improving adherence among Chinese CD patients through greater familiarity and acceptability. Thus, the MMD represents a feasible dietary model that integrates evidence-based nutritional recommendations with local dietary traditions. Given its potential to improve both adherence and clinical outcomes, this study was designed to evaluate the effects of MMD supplemented with PEN in post-surgical CD patients compared with EEN. The primary objective was to assess its impact on quality of life, while secondary objectives included evaluating disease-related characteristics and nutritional status.

## Methods

### Study design and participants

This randomised, controlled, pilot study was conducted at The Second Affiliated Hospital of Zhejiang University School of Medicine between September 2022 and June 2024. Eligible patients were individuals aged 14 years or older who had a confirmed diagnosis of CD through endoscopy, histology and imaging. The enrolled patients were in the perioperative period, defined as within 2–7 d after surgery, having undergone terminal ileal resection, partial colectomy, ileocecal resection or small bowel resection due to CD. Participants were required to be able to orally consume nutritional formulations or food, own a smartphone with the WeChat application installed and demonstrate the ability to send and receive messages via WeChat. Additionally, participants needed to be willing and able to attend scheduled postoperative follow-up visits at the study centre. Exclusion criteria included any of the following: a planned surgery within 5 weeks postoperatively; ileostomy or colostomy; complete intestinal obstruction or fibrostenosis with pre-stenotic dilatation; pregnancy or breastfeeding; planned use of biologics within 5 weeks postoperatively; planned use of corticosteroids or prednisone greater than 20 mg per day or the equivalent within 5 weeks postoperatively to maintain remission; planned use of probiotics or prebiotics for more than 1 week postoperatively; planned use of antibiotics for more than 2 weeks postoperatively; allergies to known components of enteral nutrition; a BMI of less than 14 or greater than 28 kg/m^2^; celiac disease; coexisting diabetes or other autoimmune diseases (e.g. rheumatic disease, autoimmune liver disease, psoriasis, etc.); mental illness or malignant tumours. The BMI cut-off values were chosen because a BMI below fourteen indicates severe malnutrition, requiring more intensive nutritional interventions such as EEN, rather than participation in a randomised trial^(14)^. Conversely, a BMI above twenty-eight is classified as obesity, and continuing EEN may exacerbate this condition^(17)^. Additionally, participants were excluded if they were involved in other clinical trials or had any condition that the investigator deemed made them unsuitable for participation in this study. The study was performed in accordance with the ethical principles of the Declaration of Helsinki. All participants provided written informed consent prior to the study. The study design was reviewed and approved by the Ethics Committee of Second Affiliated Hospital of Zhejiang University School of Medicine.

### Randomisation and masking

All enrolled patients were randomly assigned in a 1:1 ratio to either the MMD supplemented with PEN group or the EEN group using a computer-generated random numbers table for simple randomisation. A dietitian provided nutritional guidance to the patients based on their assigned diet 2–7 d post-surgery. Due to the nature of the intervention, blinding was not feasible; therefore, this study was conducted as an open-label trial.

### Interventions

All participants were not given any medication for treating CD, including aminosalicylates, immunosuppressants, corticosteroids, and biologics, during the 4–5 weeks dietary intervention that started on the date of surgery. Each participant completed comprehensive training on either the MMD supplemented with PEN or the EEN according to their assigned group, with the goal of achieving target energy intake of 30–35 kcal/kg·bw and target protein intake of 1·2–1·5 g/kg·bw. EEN requires patients to consume a specialised formula exclusively, with the exclusion of all solid foods and any liquids other than water. This formula is designed to provide complete nutrition, delivering all essential nutrients and energy content necessary to support healing and growth. In the MMD supplemented with PEN group, participants obtain approximately half of their energy from a specialised formula and half from food. The details regarding the food ingredients and cooking methods can be found in the online Supplementary material.

On day 2 or day 3 post-surgery, the trial protocol would commence, provided the surgeon deemed the participant suitable for oral intake or enteral nutrition. At this time, baseline data were collected. During the trial, all participants followed the EEN or MMD supplemented with PEN diets under the supervision of a dietitian based on their assigned groups (online Supplementary material). Each participant was instructed to eat their meals alone and to document the cooked weight of all foods ingested at each meal, along with the corresponding raw-to-cooked ratios. Dietitians utilised the recorded cooked weights and raw-to-cooked ratios to ascertain the raw weight of each food item, which was subsequently input into Nutritional Calculator software to estimate daily energy and nutrient intakes. The net weight of each food ingredient, both prior to and following cooking, was documented to facilitate the calculation of the raw-to-cooked ratio. The raw/cooked ratio was calculated as follows:

$$Ratio=\frac{Weight\;of\;edible\;portion\;of\;each\;food\;ingredient\;before\;cooking}{Weight\;of\;cooked\;food\;ingredient\;after\;cooking}.$$

Dietary intake data were systematically recorded on a daily basis in an electronic food diary and subsequently transmitted to the dietitian for analysis via WeChat. The dietitian maintained continuous communication with participants between 17.00 and 22.00 each day to provide feedback and guidance, ensuring accurate monitoring and support of dietary adherence. Based on this information and nutrient analysis, the dietitian provided personalised dietary instructions for the following day, aiming to meet a daily energy target. Like the MMD supplemented with PEN group, participants in the EEN group also reported their daily consumption to the dietitian.

The programme lasted 4–5 weeks for all participants. This duration was selected for two main reasons: firstly, participants were scheduled to begin pharmacological treatments in the subsequent phase of care; secondly, considering that early postoperative dietary intervention may influence patients’ long-term food preferences, a 21-day period is generally sufficient for establishing dietary habits. If feasible in future studies, longer intervention periods may be explored.

### Outcome measurement

The primary outcome of the study was quality of life, measured using the IBDQOL-22^(18)^ at the end of the intervention. The IBDQOL-22 has twenty-two items, which assess the quality of life of patients with CD in four dimensions: bowel symptoms and their influences (six items), symptoms and discomfort (five items), emotional function (six items) and social functions (five items). The total score ranges from twenty-two to 110, and higher scores indicate a better quality of life. The IBDQOL-22 demonstrated good internal consistency in CD, with Cronbach’s *α* values ranging from 0·76 to 0·89, and excellent test–retest reliability, with intraclass correlation coefficients of 0·72 to 0·90^(18)^.

Secondary outcomes included nutritional status and disease-related characteristics assessed at the end of the intervention. Nutritional status indicators included (g/l), erythrocyte (10^12^/l), creatinine (μmol/l) and albumin (g/l). Disease-related characteristics were comprehensively evaluated using clinical indices, symptom scales and laboratory markers. Clinical disease activity was assessed with the Harvey–Bradshaw Index (HBI)^(19)^ and the Crohn’s Disease Activity Index^(20)^, both validated measures of disease severity. Gastrointestinal symptoms were captured using the patient-reported Gastrointestinal Symptom Rating Scale^(21)^. Laboratory markers included platelet count, C-reactive protein and erythrocyte sedimentation rate to reflect systemic inflammation, while faecal calprotectin was measured as a non-invasive indicator of intestinal mucosal inflammation and treatment response. Physician-assessed poor adherence was operationally defined as the fulfilment of at least one of the following criteria: (1) refusal to comply with the prescribed nutritional guidance provided at the time of study enrolment or (2) failure to submit the electronic food diary for more than 10 % of the total study duration (online Supplementary material).

### Power calculation

A sample size estimation was not performed since the purpose of this study was to evaluate the feasibility and potential effects of the intervention to generate preliminary data for future large-scale randomised controlled trials. There was also no previous data available on the difference between IBDQOL-22 scores of the MMD supplemented with PEN and EEN groups. A total of forty-two to fifty-three participants were expected based on the average hospitalisation rate at the centre of investigation during the 21-month recruiting period, assuming 4–5 CD patients per month and an inclusion rate of 50 %.

### Statistical analysis

Data analysis was conducted using SPSS software (Version 27, USA) on a modified intention-to-treat basis. Patients who experienced disease worsening, developed new complications or required drug treatment for CD were withdrawn from the trial, and their data were considered a failure of the intervention and excluded from the analysis. Categorical variables were analysed using the χ^2^ test. Continuous variables were reported as means (sd) or medians (interquartile range). Normally distributed data underwent analysis using Student’s *t* test, while skewed data were analysed using the Mann–Whitney U test or Kruskal–Wallis H test as appropriate. P-values less than 0·05 were deemed statistically significant.

## Results

### Patient enrolment and baseline information

**Figure 1.**
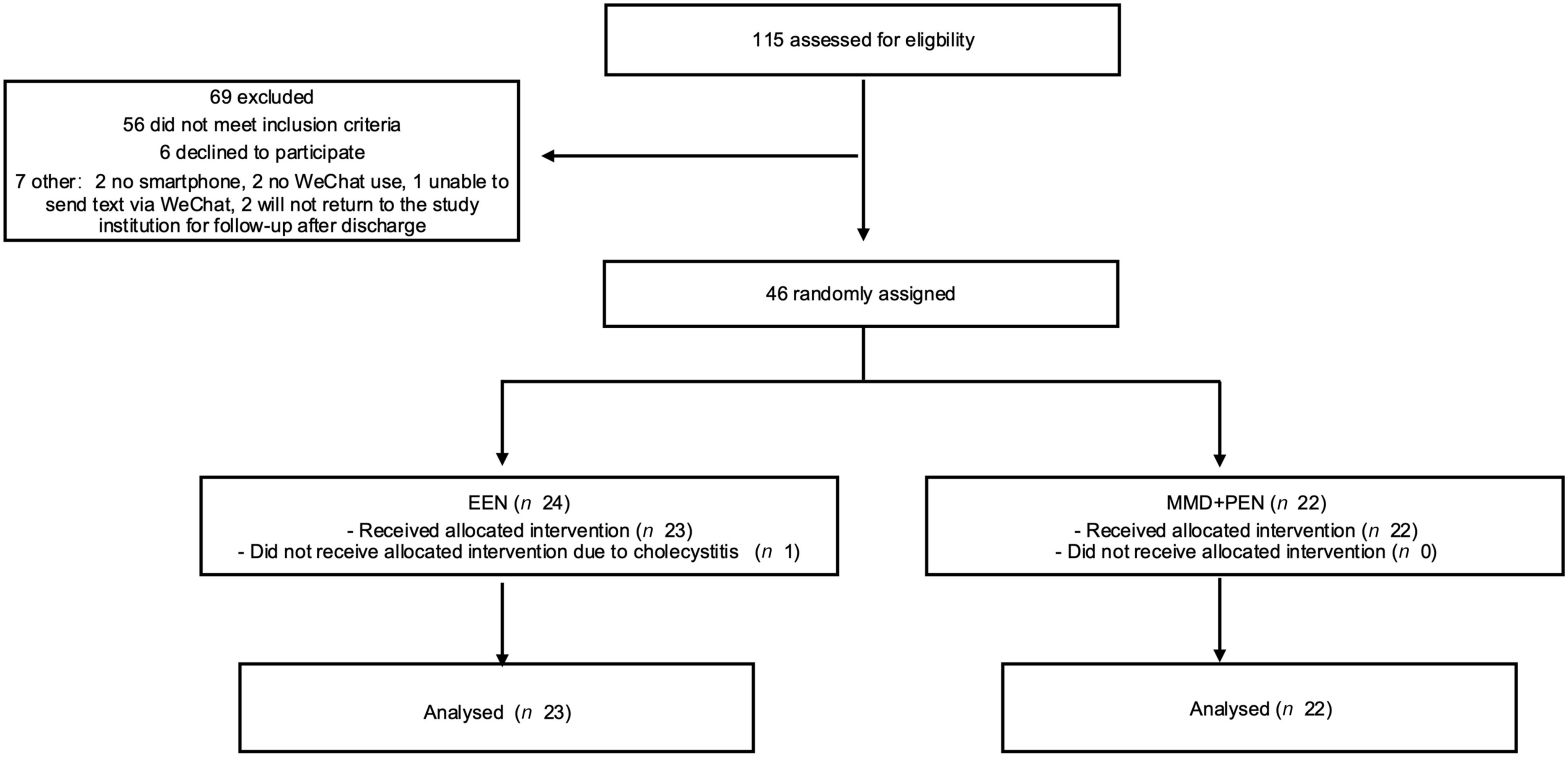
Study enrolment and allocation.EEN, Exclusive enteral nutrition; MMD, Modified Mediterranean diet; PEN, Partial enteral nutrition.

A total of 115 patients were recruited and assessed for eligibility for the study, of whom forty-six were randomly assigned to the EEN group (*n* 24) and MMD supplemented with PEN group (*n* 22) (Figure 1). One patient in the EEN group did not receive allocated intervention due to cholecystitis and was thus excluded from the analysis. A total of twenty-three patients in the EEN group and twenty-two patients in the MMD supplemented with PEN group adhered to the study protocol, received complete intervention and were included in the analysis. The mean age of the EEN and MMD supplemented with PEN groups was 31·91 (sd 8·26) years and 35·73 (sd 9·68), respectively. The proportion of males was 54·5 % and 45·5 % (*P* = 0·445). Mean BMI was 19·9 (sd 3·2) in the EEN group and 19·6 (sd 2·4) in the MMD supplemented with PEN group (*P* = 0·697). Median disease duration was 3·0 and 2·5 years, respectively (*P* = 0·471). The median baseline IBDQOL scores of the EEN and MMD supplemented with PEN groups were comparable (77·00 (sd 12·61) *v*. 72·27 (sd 13·42), *P* = 0·230). A higher proportion of patients in the EEN group had perianal disease compared with the MMD supplemented with PEN group (47·8 % *v*. 18·2 %, *P* = 0·035). This imbalance in baseline perianal involvement may potentially influence clinical outcomes, as perianal disease is often associated with more severe disease course and may affect nutritional status, response to dietary interventions and quality-of-life measures. Therefore, caution is warranted when interpreting intervention effects, and this factor should be considered (Table 1).

**Table 1.** Baseline characteristics Table 1 long description.

Characteristics	EEN (*n* 23)		MMD supplemented with PEN (*n* 22)		*P*
	Mean	sd	Mean	sd	
Age at randomisation, years, mean (sd)	31·91	8·26	35·73	9·68	0·162
	*n*	%	*n*	%	
Gender, *n* (males %)	18	54·5	15	45·5	0·445
Education, *n* (%)					0·088
High school or below	2	8·7	6	27·3	
Undergraduate	17	73·9	11	50	
Master’s degree or above	2	8·7	0	0	
Other	2	8·7	5	22·7	
Source of funds for medical expenses, *n* (%)					0·953
Government Funding	0	0	0	0	
Medical Insurance	20	87	19	86·4	
Commercial Insurance	0	0	0	0	
Self-pay	0	0	0	0	
Other	3	13	3	13·6	
Smoking status, *n* (%)					0·524
Never	22	95·7	20	90·9	
Past	1	4·3	2	9·1	
Current	0	0	0	0	
	Mean	sd	Mean	sd	
BMI, kg/m^2^, mean (sd)	19·92	3·19	19·59	2·40	0·697
	Median	IQR	Median	IQR	
Age at diagnosis, years, median (IQR)	25	23–28	30·5	24–36	0·077
Disease duration, years, median (IQR)	3	1–10	2·5	1–6	0·471
	*n*	%	*n*	%	
Montreal classification					
Crohn’s disease location, *n* (%)					0·557
Ileal	9	39·1	12	54·5	
Colonic	2	8·7	1	4·5	
Ileocolonic	12	52·2	9	40·9	
CD behaviour, *n* (%)					0·862
Nonstricturing, nonpenetrating	0	0	0	0	
Stricturing	10	43·5	9	40·9	
Penetrating	13	56·5	13	59·1	
Perianal disease, *n* (%)	11	47·8	4	18·2	0·035
Extraintestinal manifestations, *n* (%)	1	4·3	1	4·5	0·368
	Median	IQR	Median	IQR	
Start time of flatus passage (post-surgical day), median (IQR)	3	2–4	3	3–4	0·330
Start time of defecation (post-surgical day), median (IQR)	4	3–5	4	3–5	0·597
Days of parenteral nutrition					
Mean	8·22		7·55		0·317
sd	2·60		1·77		
Start time of enteral nutrition (post-surgical day), median (IQR)	4	4–5	4	3–6	0·676
	*n*	%	*n*	%	
History of surgery, *n* (%)					0·098
Surgery excluding intestinal resection	17	73·9	11	50	
No surgery	6	26·1	11	50	
	Median	IQR	Median	IQR	
Preoperative EEN time, days, median (IQR)	52	30–60	41	26·25–60	0·655
IBDQOL^*^					
Mean	77·00		72·27		0·230
sd	12·61		13·42		
HBI^*^, median (IQR)	3	1–4	3	1–5	0·395
	*n*	%	*n*	%	
HBI, *n* (%)					0·279
≤ 4	19	82·6	14	63·6	
5–8	4	17·4	7	31·8	
≥ 9	0	0	1	4·5	
CDAI^*^					
Mean	162·37		167·21		0·790
sd	57·03		64·20		
CDAI, *n* (%)					0·604
< 150	10	43·5	7	31·8	
150–220	10	43·5	10	45·5	
221–450	3	13	5	22·7	
> 450	0	0	0		
GSRS^*^					
Median	22		25		0·155
IQR	20–25		21–28		
	Mean	sd	Mean	sd	
Hb, g/l, mean (sd)	106·70	18·74	104·73	15·04	0·700
Erythrocyte,10^12^/l, mean (sd)	3·69	0·52	3·62	0·53	0·652
	Median	IQR	Median	IQR	
PLT, 10^9^/l, median (IQR)	199	174–243	223	192·75–248	0·307
CRP, mg/l, median (IQR)	24·3	12·0–37·5	23·85	15·93–34·53	0·937
ESR, mm/h, median (IQR)	22	5–36	19	7–34·25	0·838
FC, μg/g, median (IQR)	666·30	389·94–1561·74	884·73	556·20–1410·95	0·451
	Mean	sd	Mean	sd	
ALB, g/l, mean (sd)	35·55	3·70	37·13	3·37	0·143
Cr, μmol/l, mean (sd)	57·59	13·03	54·36	11·30	0·379

EEN, Exclusive enteral nutrition; MMD, Modified Mediterranean diet; PEN, Partial enteral nutrition; CD, Crohn’s disease; IQR, Interquartile range; IBDQOL, Inflammatory bowel disease quality-of-life questionnaire; HBI, Harvey–Bradshaw Index score; CDAI, Crohn’s Disease Activity Index; GSRS, Gastrointestinal Symptom Rating Scale; PLT, platelet count; CRP, C-reactive protein; FC, Faecal calprotectin; ESR, Erythrocyte sedimentation rate; ALB, albumin; Cr, Creatinine.

* Data missing for one patient.

### Primary and secondary outcomes

**Figure 2.**
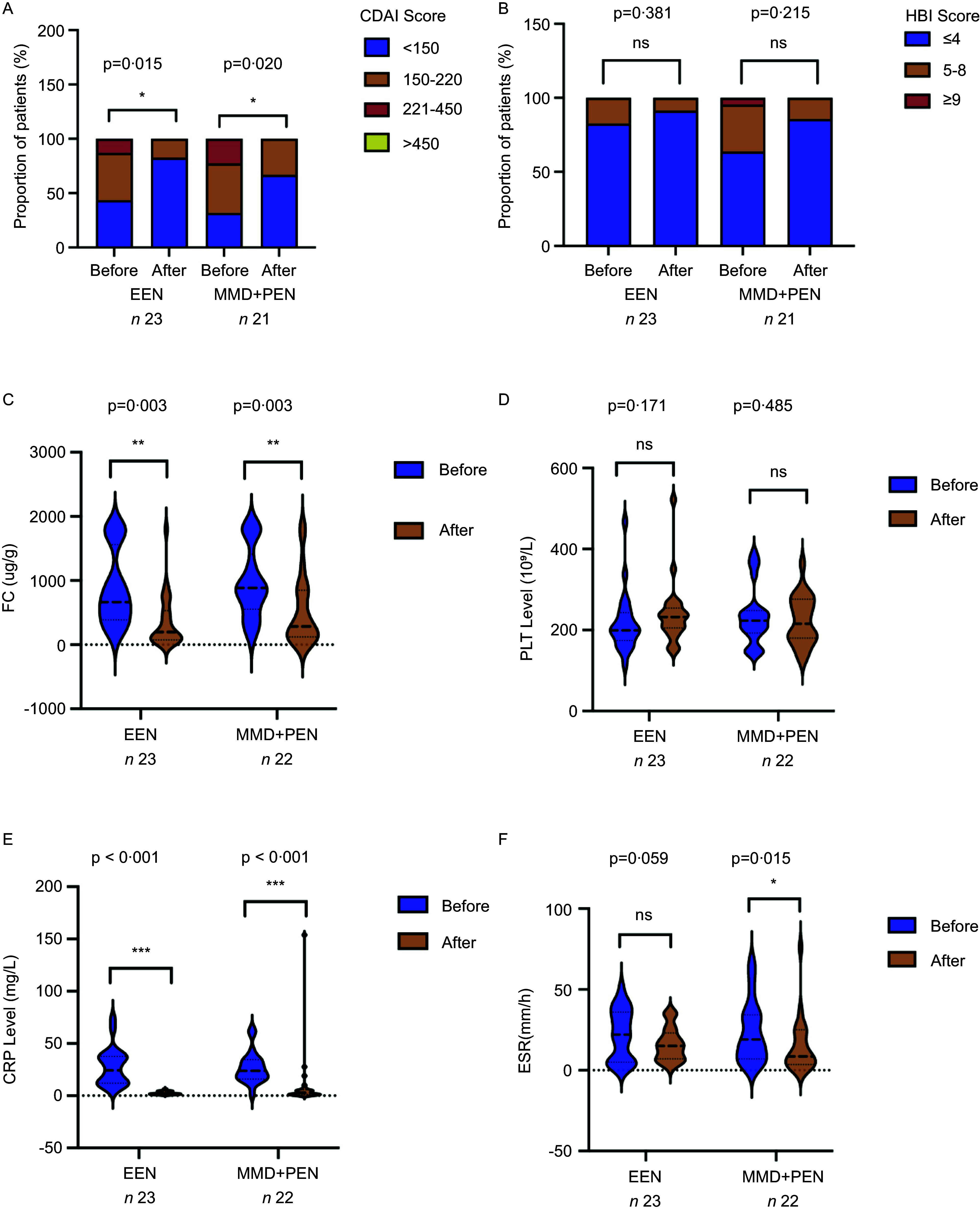
Quality of life and nutritional status in the EEN and MMD supplemented with PEN groups. CDAI, Crohn’s Disease Activity Index; HBI, Harvey–Bradshaw Index score; EEN, Exclusive enteral nutrition; MMD, Modified Mediterranean diet; PEN, Partial enteral nutrition; FC, Faecal calprotectin; PLT, platelet count; CRP, C-reactive protein; ESR, Erythrocyte sedimentation rate. Ns, non-significant; *P < 0·05, **P < 0·01, ***P < 0·001.

At the end of the intervention, the EEN and MMD supplemented with PEN groups achieved similar IBDQOL scores (88·43 (sd 9·17) *v*. 87·57 (sd 7·38), *P* = 0·734) (Table 2). Patients in both groups showed significant improvement in IBDQOL scores after intervention (*P* < 0·001) (Figure 2). There was also no significant difference in all four aspects of the IBDQOL between the EEN and MMD supplemented with PEN groups (*P* > 0·05) (Table 3).

**Table 2.** Primary and key secondary outcomes Table 2 long description.

	EEN (*n* 23)		MMD supplemented with PEN (*n* 22)		*P*
	Mean	sd	Mean	sd	
Primary outcome					
IBDQOL	88·43	9·17	87·57	7·38	0·734
	Median	IQR	Median	IQR	
Secondary outcomes					
HBI, median (IQR)	1	0–3	3	0–4	0·463
	*n*	%	*n*	%	
HBI, *n* (%)					0·560
≤ 4	21	91·3	18	85·7	
5–8	2	8·7	3	14·3	
≥ 9	0	0	0	0	
CDAI					
Mean	108·07		115·47		0·625
sd	48·17		51·61		
CDAI, *n* (%)					0·223
< 150	19	82·6	14	66·7	
150–220	4	17·4	7	33·3	
221–450	0	0	0	0	
> 450	0	0	0	0	
	Median	IQR	Median	IQR	
GSRS	23	19–26	22	20·5–26·5	0·556
Hb, g/l					
Mean	126·65		126·41		0·952
sd	11·96		15·09		
Erythrocyte,10^12^/l					
Mean	4·36		4·37		0·954
sd	0·42		0·56		
PLT, 10^9^/l, median (IQR)	232	205–254	215	180–276·25	0·674
CRP, mg/l, median (IQR)	2	1·15–4·00	2·75	1·05–5·73	0·481
ESR, mm/h, median (IQR)	15	7–23	8·5	3·5–25	0·246
FC, μg /g, median (IQR)	198·53	76·01–531·07	283·87	121·29–847·18	0·242
	Mean	sd	Mean	sd	
ALB, g/l, mean (sd)	45·74	2·40	44·88	3·15	0·308
Cr, μmol/l, mean (sd)	66·46	11·94	61·46	9·40	0·127

EEN, Exclusive enteral nutrition; MMD, Modified Mediterranean diet; PEN, Partial enteral nutrition; IQR, Interquartile range; IBDQOL, Inflammatory bowel disease quality-of-life questionnaire; HBI, Harvey–Bradshaw Index score; CDAI, Crohn’s Disease Activity Index; GSRS, Gastrointestinal Symptom Rating Scale; PLT, Platelet count; CRP, C-reactive protein; FC, Faecal calprotectin; ESR, Erythrocyte sedimentation rate; ALB, Albumin; Cr, Creatinine.

**Table 3.** Inflammatory bowel disease quality-of-life questionnaire at baseline and after the intervention Table 3 long description.

	Baseline					End of the intervention				
	EEN (*n* 23)		MMD supplemented with PEN (*n* 22)			EEN (*n* 23)		MMD supplemented with PEN (*n* 21)		*P*
	Mean	sd	Mean	sd	*P*	Mean	sd	Mean	sd	
Bowel symptoms and their influences	23·87	3·68	23·23	3·19	0·358	27·52	2·17	25·71	2·78	0·431
Symptoms and discomfort	17·61	3·96	16·45	5·23	0·126	20·78	2·65	20·33	2·33	0·577
Emotional function	19·87	4·61	18·14	4·02	0·632	22·61	4·31	23·43	4·07	0·914
Social functions	15·65	4·38	14·45	3·81	0·294	17·52	2·66	18·10	2·28	0·948

EEN, Exclusive enteral nutrition; MMD, Modified Mediterranean diet; PEN, Partial enteral nutrition.

At the end of the intervention, there were no significant differences between the EEN and MMD + PEN groups in terms of HBI (1 (0, 3) *v*. 3 (0, 4), *P* = 0·463), Crohn’s Disease Activity Index (108·07 (sd 48·17) *v*. 115·47 (sd 51·61), *P* = 0·625) and Gastrointestinal Symptom Rating Scale (23 (19, 26) *v*. 22 (20·5, 26·5), *P* = 0·556) scores, or other disease-related characteristics, including platelet count, C-reactive protein, erythrocyte sedimentation rate and faecal calprotectin. Similarly, Hb, erythrocyte, creatinine and albumin levels were comparable between the two groups (Table 2).

**Figure 3.**
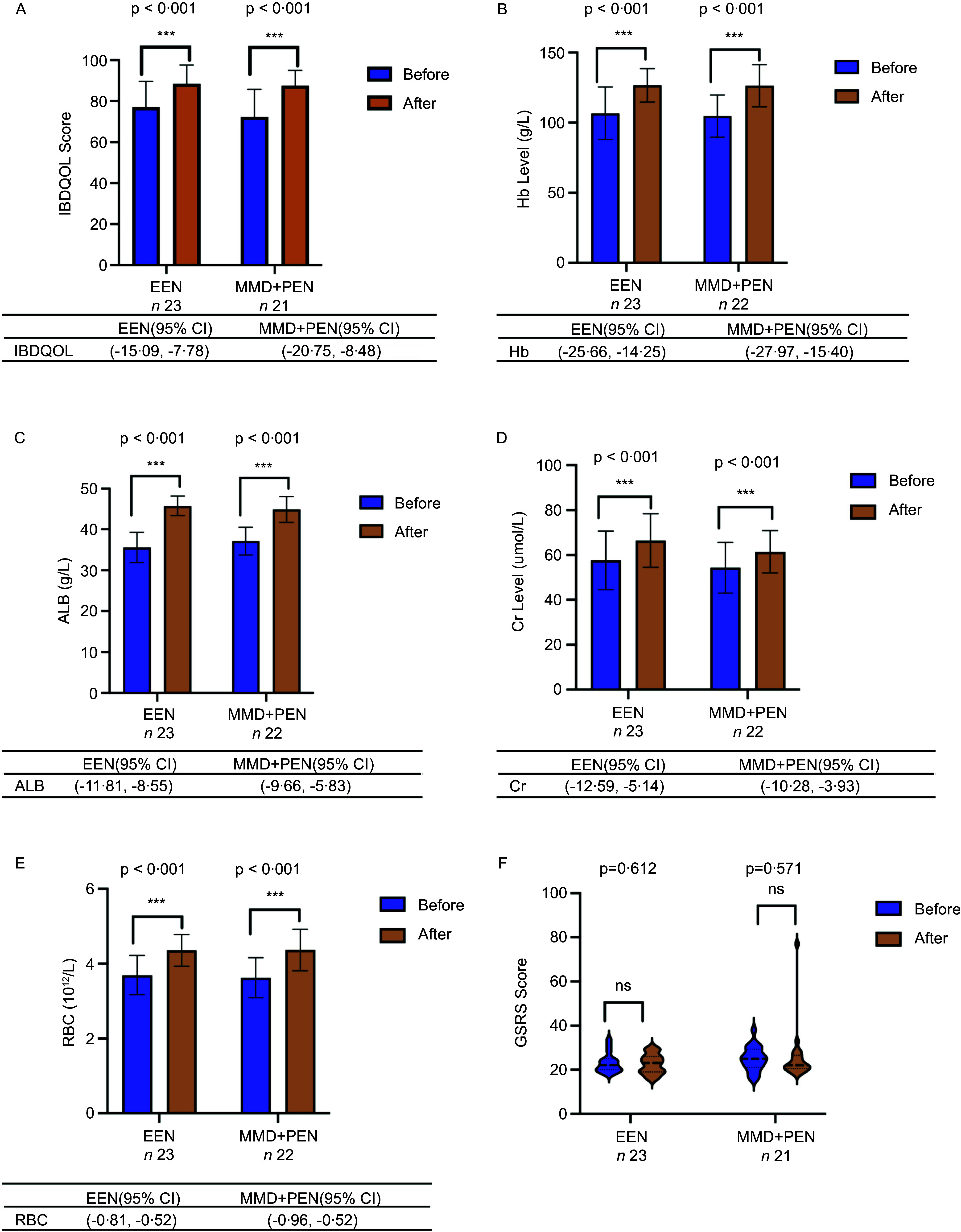
Disease-related characteristics in the EEN and MMD supplemented with PEN groups. IBDQOL, Inflammatory bowel disease quality-of-life questionnaire; EEN, Exclusive enteral nutrition; MMD, Modified Mediterranean diet; PEN, Partial enteral nutrition; ALB, albumin; Cr, Creatinine; GSRS, Gastrointestinal Symptom Rating Scale. Ns, non-significant; *P < 0·05, **P < 0·01, ***P < 0·001.

Within-group comparisons showed that, although HBI and Gastrointestinal Symptom Rating Scale scores did not change significantly, the number of patients with HBI > 5 decreased in both groups after intervention (an HBI score greater than five generally indicates active CD, whereas a score below five is consistent with clinical remission). Both EEN and MMD + PEN groups exhibited increases in creatinine, Hb, erythrocyte and albumin levels (Figure 2), along with decreases in C-reactive protein and faecal calprotectin levels (Figure 3) relative to baseline. Additionally, the MMD + PEN group demonstrated a reduction in erythrocyte sedimentation rate levels at the end of the intervention (Figure 3).

### Safety

One patient in the EEN group (4·2 %) had cholecystitis and was withdrawn from the study. No other adverse events or serious adverse events were observed or reported during the study.

### Patient adherence

Participant adherence to the study protocol was evaluated. Based on the predefined criteria, adherence in both study groups was determined to be 100 %, with no participants classified as exhibiting poor adherence. This favourable outcome is likely attributable to the comprehensive initial training and the regular communication with a dietitian to review daily food and nutritional formulation intake, which may have enhanced participant engagement and self-awareness in nutritional management.

## Discussion

In this pilot study, we demonstrated that MMD supplemented with PEN achieved comparable improvements in overall quality of life to EEN in post-surgical CD patients. Both groups achieved similar IBDQOL scores. Furthermore, MMD supplemented with PEN and EEN yielded comparable outcomes in disease-related characteristics and nutritional status at the end of the study, which were significantly improved compared with baseline. Post-surgical CD patients often face substantial dietary restrictions, which can compromise adherence and limit the long-term utility of nutritional interventions. Remarkably, in this trial, all participants completed the study, suggesting that the real-world feasibility and cultural adaptability of the MMD may help mitigate adherence challenges typically encountered with restrictive regimens such as EEN. Notably, the anti-inflammatory efficacy of MMD with PEN was confirmed by significant reductions in C-reactive protein and faecal calprotectin levels, achieved without serious adverse events. These findings suggest that MMD supplemented with PEN may be a promising diet that delivers adequate nutrition and effectively reduces inflammation in post-surgical CD patients, with the potential for wider application in Chinese population.

Immunosuppression is still the primary treatment for CD, despite its potential side effects and high costs. In contrast, dietary therapy offers a promising alternative for managing CD, particularly for patients who are not suitable candidates for immunosuppressive therapy within 4–5 weeks after surgery, and it carries a relatively low risk of side effects. Due to the potential inflammatory factors present in normal diets, CD patients are often over-concerned about diet control, leading to strict and unnecessary dietary restrictions to maintain remission and prevent recurrence^(22)^. This may lead to malnutrition, additional stress, disordered eating, as well as diminished emotional and social well-being, ultimately reducing their quality of life. While EEN could provide sufficient nutrition with low or no side effects, it is non-sustainable^(23)^ and has poor palatability that induces poor compliance and adherence^(9)^, limiting its efficacy. Moreover, the re-introduction of a habitual diet after EEN may induce a rebound increase in inflammatory indices in CD patients^(6,7)^. Alternative dietary approaches, such as CDED^(6,11,24)^, Mediterranean diet^(25)^, solid food diet^(26)^ and low fermentable oligosaccharides, disaccharides, monosaccharides and polyols diet^(27)^, have shown efficacy in several studies in different populations. Importantly, CDED and similar diets may provide long-term protection by alternating the gut microbiota^(28)^, with high sustainability due to their relatively low cost and high rate of compliance. However, they may be less suitable for Chinese CD patients due to different dietary habits and limited ingredient accessibility. In this study, we introduced MMD supplemented with PEN to cater to Chinese CD patients’ dietary habits. MMD supplemented with PEN has a similar nutritional composition to CDED and the Mediterranean diet but is based on food ingredients that are easily accessible in China. MMD supplemented with PEN also emphasises mild cooking methods, including steaming, boiling and low-temperature frying with a limited amount of oil. These techniques may reduce inflammatory triggers in meals while promoting nutrient absorption, providing a culturally appropriate and effective dietary solution for Chinese CD patients. Indeed, MMD supplemented with PEN significantly improved the quality of life of post-surgical patients, reaching similar clinical outcomes compared with EEN. It should also be noted that patients receiving drug treatment were excluded from this study, eliminating the potential biases introduced by drug treatment in our study. The contribution of PEN should also be acknowledged in interpreting these results. As a controlled and nutrient-dense supplement, PEN may help compensate for nutritional gaps inherent in the MMD, which could have supported the improvements observed in both clinical and biochemical outcomes. Therefore, the comparable efficacy of MMD with PEN to EEN may be explained by the synergistic effect of an anti-inflammatory dietary pattern combined with the consistent nutrient delivery provided by PEN.

Another significant advantage of the MMD supplemented with PEN is its emphasis on enhanced patient monitoring and education. In this study, all patients in the MMD supplemented with PEN group received initial training in the first week and maintained regular communication with a dietitian to discuss their daily food consumption. These interactions may have strengthened their engagement and improved their awareness of dietary control. It has been shown that patient-centred care, active participation and shared decision-making are critical in long-term CD management^(29)^. The European Crohn’s and Colitis Organisation consensus also agreed that ‘optimising quality of care in inflammatory bowel disease involves information and education’^(30)^. Such active involvement may also positively affect the patients’ psychological state and adherence by developing a sense of empowerment and control. Furthermore, patient education on diet preparation and nutrition may bring long-lasting benefits even beyond the intervention programme and contribute to long-term CD remission.

There are several limitations with this study. Firstly, this pilot, open-label, single-centred study only included a limited number of post-surgical CD patients, and the findings may be subject to bias and have limited generalisability. Since no prior data were available on the difference between the MMD + PEN and EEN groups, no *a priori* sample size estimation was feasible. Consequently, the small sample size may have reduced the statistical power to detect subtle between-group differences, which could partly explain the lack of statistically significant findings for quality-of-life outcomes. A well-powered study is required to validate the efficacy of MMD supplemented with PEN in a larger and more diverse patient population. Secondly, the intervention lasted only 4–5 weeks as participants were scheduled to initiate pharmacological treatments in the subsequent phase of care. This short duration may have restricted the potential to detect longer-term effects, including sustained improvements in quality of life and the ability of the MMD supplemented with PEN to induce or maintain remission. Thirdly, most participants were in remission or had mildly active disease at enrolment, which limits conclusions regarding the efficacy of MMD supplemented with PEN in patients with more severe disease activity. Finally, endoscopic evaluation was not performed at the end of the intervention, preventing confirmation of mucosal healing. Larger, adequately powered and longer-term studies are warranted to validate these findings and to further clarify the role of MMD supplemented with PEN in post-surgical CD management.

In summary, this study is the first prospective clinical trial to investigate the effects of MMD supplemented with PEN on post-surgical CD patients in China. Our findings suggest that MMD supplemented with PEN could improve the quality of life, nutritional status and disease-related characteristics in these patients, reaching outcomes comparable to those of EEN. These results suggest that MMD supplemented with PEN may serve as a promising dietary plan for the management of post-surgical CD patients. Future large-scale clinical trials are needed to validate the efficacy and safety of MMD supplemented with PEN.

## Supporting information

Huang et al. supplementary material 1 Huang et al. supplementary material

Huang et al. supplementary material 2 Huang et al. supplementary material

Huang et al. supplementary material 3 Huang et al. supplementary material

## Supplementary material

For supplementary material/s referred to in this article, please visit https://doi.org/10.1017/S0007114526106588

## Table 1 Long description

A table comparing baseline characteristics of two patient groups, EEN and MMD supplemented with PEN. The table has 42 rows and 11 columns. Column headers are Characteristics, EEN (n 23), and MMD supplemented with PEN (n 22). Row labels include Age at randomisation, years, mean (sd); Gender, n (male %); Education, n (%); High school or below; Undergraduate; Master's degree or above; Other; Source of funds for medical expenses, n (%); Government Funding; Commercial Insurance; Self-pay; Other; Smoking status, n (%); Never; Past; Current; BMI, kg/m^2, mean (sd); Age at diagnosis, years, median (IQR); Disease duration, years, median (IQR); Montreal classification; Crohn's disease location, n (%); Real; Colonic; Ileocolonic; CD behaviour, n (%); Non-stricturing, non-penetrating; Stricturing; Penetrating; Perianal disease, n (%); Extranintestinal manifestations, n (%); Start time of flatus passage (post-surgery), median (IQR); Start time of defecation (post-surgery), median (IQR); Days of parenteral nutrition; Start time of enteral nutrition (post-surgery), median (IQR); History of surgery, n (%); No surgery; Surgery excluding intestinal resection; Surgery including intestinal resection; Preoperative EEN time, days, median (IQR); IBDQOL, mean (sd); Hb, g/L, median (IQR); Hb, n (%); Albumin, g/L, median (IQR); CRP, mg/L, median (IQR); ESR, mm/h, median (IQR). Each row provides specific values for each characteristic in both groups. Notable trends include differences in smoking status, perianal disease, and preoperative EEN time between the two groups.

Navigate back to Table 1.

## Table 2 Long description

The table compares primary and secondary outcomes between two groups: EEN (n = 23) and MMD supplemented with PEN (n = 22). It has 16 rows and 8 columns. The columns are labeled as follows: Primary outcome, IBDQOL (Mean, SD, Median, IQR), Secondary outcomes, HBI (median, IQR, n, %), CDAI (Mean, SD, n, %), GSRS (Median, IQR), Hb (Mean, SD), Erythrocyte (Mean, SD), PLT (median, IQR), CRP (median, IQR), ESR (median, IQR), FC (median, IQR), ALB (Mean, SD), Cr (Mean, SD). The table presents the mean, standard deviation (SD), median, interquartile range (IQR), and number (n) with percentage (%) for various outcomes. Row 1: Primary outcome. Row 2: IBDQOL Mean, EEN 88.43, SD 9.17, MMD 87.57, SD 7.38, P 0.734. Row 3: IBDQOL Median, EEN 0.3, IQR 0-3, MMD 0-4, P 0.463. Row 4: HBI n (%), EEN 21 (91.3%), MMD 18 (85.7%), P 0.560. Row 5: HBI 5-8, EEN 2 (8.7%), MMD 3 (14.3%). Row 6: HBI >= 9, EEN 0 (0%), MMD 0 (0%). Row 7: CDAI Mean, EEN 108.07, SD 48.17, MMD 115.47, SD 51.61, P 0.625. Row 8: CDAI n (%), EEN 19 (82.6%), MMD 14 (66.7%), P 0.223. Row 9: CDAI 150-220, EEN 4 (17.4%), MMD 7 (33.3%). Row 10: CDAI >= 450, EEN 0 (0%), MMD 0 (0%). Row 11: CDAI Median, EEN 19-26, MMD 20.5-26.5, P 0.556. Row 12: Hb Mean, EEN 126.65, SD 11.96, MMD 126.41, SD 15.09, P 0.952. Row 13: Erythrocyte Mean, EEN 4.36, SD 0.42, MMD 4.37, SD 0.56, P 0.954. Row 14: PLT Median, EEN 232, IQR 205-254, MMD 215, IQR 180-276.25, P 0.674. Row 15: CRP Median, EEN 2, IQR 1.15-4.00, MMD 2.75, IQR 1.05-5.73, P 0.481. Row 16: ESR Median, EEN 15, IQR 7-23, MMD 8.5, IQR 3.5-25, P 0.246. Row 17: FC Median, EEN 198.53, IQR 76.01-531.07, MMD 283.87, IQR 121.29-847.18, P 0.242. Row 18: ALB Mean, EEN 45.74, SD 2.40, MMD 44.88, SD 3.15, P 0.308. Row 19: Cr Mean, EEN 66.46, SD 11.94, MMD 61.46, SD 9.40, P 0.127.

Navigate back to Table 2.

## Table 3 Long description

A table comparing quality-of-life scores for inflammatory bowel disease patients at baseline and after the intervention. The table has four rows and eight columns. The columns are labeled as follows: Baseline EEN (n 23) Mean, Baseline EEN (n 23) SD, Baseline MMD supplemented with PEN (n 22) Mean, Baseline MMD supplemented with PEN (n 22) SD, Baseline P, End of the intervention EEN (n 23) Mean, End of the intervention EEN (n 23) SD, End of the intervention MMD supplemented with PEN (n 21) Mean, End of the intervention MMD supplemented with PEN (n 21) SD, End of the intervention P. The rows are labeled as follows: Bowel symptoms and their influences, Symptoms and discomfort, Emotional function, Social functions. Row 1: Bowel symptoms and their influences, 23.87, 3.68, 23.23, 3.19, 0.358, 27.52, 2.17, 25.71, 2.78, 0.431. Row 2: Symptoms and discomfort, 17.61, 3.96, 16.45, 5.23, 0.126, 20.78, 2.65, 20.33, 2.33, 0.577. Row 3: Emotional function, 19.87, 4.61, 18.14, 4.02, 0.632, 22.61, 4.31, 23.43, 4.07, 0.914. Row 4: Social functions, 15.65, 4.38, 14.45, 3.81, 0.294, 17.52, 2.66, 18.10, 2.28, 0.948.

Navigate back to Table 3.
