# Supplementary material for: Effect of modified Mediterranean diet supplemented with partial enteral nutrition in post-surgical patients with Crohn’s disease: a pilot clinical trial
Source: Br J Nutr. 2026 Feb 27;135(12):1315–25. doi: 10.1017/S0007114526106588 (PMC13423520; doi:10.1017/S0007114526106588)
Supplement: Huang et al. supplementary material 1 — Huang et al. supplementary material [file S0007114526106588sup001.docx]

**Physician's assessment of compliance: Poor adherence was defined as meeting at least one of the following criteria: (1) refusal to adhere to the assigned nutritional guidance provided at study enrollment; or (2) failure to submit the electronic food diary for more than 10% of the total study days.**

**Digital Food Diary Template**

| Date | Location | Food type, weight, and cooking methods | Gastrointestinal reactions (such as abdominal pain, diarrhea, bloating, etc.) | Overall feeling after meal (poor, fair, good) | Bowel Movements (frequency, consistency) | Amount of enteral formula/liquid consumed | Other |
| --- | --- | --- | --- | --- | --- | --- | --- |
|  |  |  |  |  |  |  |  |
|  |  |  |  |  |  |  |  |
