# Supplementary material for: Effect of modified Mediterranean diet supplemented with partial enteral nutrition in post-surgical patients with Crohn’s disease: a pilot clinical trial
Source: Br J Nutr. 2026 Feb 27;135(12):1315–25. doi: 10.1017/S0007114526106588 (PMC13423520; doi:10.1017/S0007114526106588)
Supplement: Huang et al. supplementary material 2 — Huang et al. supplementary material [file S0007114526106588sup002.docx]

Gastrointestinal Symptom Rating Scale

| Did you have the following symptoms in the past week? | Score | | | | | | |
| --- | --- | --- | --- | --- | --- | --- | --- |
|  | Not at all | Slightly | A little | Moderately | Fairly obvious discomfort | Relatively severe | Extremely severe |
| Pain or discomfort in upper abdomen | 1 | 2 | 3 | 4 | 5 | 6 | 7 |
| Heartburn | 1 | 2 | 3 | 4 | 5 | 6 | 7 |
| Acid reflux | 1 | 2 | 3 | 4 | 5 | 6 | 7 |
| Hunger pain | 1 | 2 | 3 | 4 | 5 | 6 | 7 |
| Nausea | 1 | 2 | 3 | 4 | 5 | 6 | 7 |
| Abdominal rumbling | 1 | 2 | 3 | 4 | 5 | 6 | 7 |
| Abdominal distension | 1 | 2 | 3 | 4 | 5 | 6 | 7 |
| Throat discomfort | 1 | 2 | 3 | 4 | 5 | 6 | 7 |
| Bad breath | 1 | 2 | 3 | 4 | 5 | 6 | 7 |
| Abnormal smell in urine | 1 | 2 | 3 | 4 | 5 | 6 | 7 |
| Constipation | 1 | 2 | 3 | 4 | 5 | 6 | 7 |
| Diarrhea | 1 | 2 | 3 | 4 | 5 | 6 | 7 |
| Loose stools | 1 | 2 | 3 | 4 | 5 | 6 | 7 |
| Dry and hard stools | 1 | 2 | 3 | 4 | 5 | 6 | 7 |
| Urgent need for defecation | 1 | 2 | 3 | 4 | 5 | 6 | 7 |
| Feeling of incomplete evacuation | 1 | 2 | 3 | 4 | 5 | 6 | 7 |
| Total |  | | | | | | |
