# Supplementary material for: Effect of modified Mediterranean diet supplemented with partial enteral nutrition in post-surgical patients with Crohn’s disease: a pilot clinical trial
Source: Br J Nutr. 2026 Feb 27;135(12):1315–25. doi: 10.1017/S0007114526106588 (PMC13423520; doi:10.1017/S0007114526106588)
Supplement: Huang et al. supplementary material 3 — Huang et al. supplementary material [file S0007114526106588sup003.docx]

| **Food Choices** | **Allowed Foods** |
| --- | --- |
|  | Rice-based foods: white rice porridge, sweet potato porridge, pumpkin porridge, rice, and rice cakes.  Wheat-based foods: noodles, wontons, dumplings, and steamed buns.  Millet-based foods: millet porridge. |
|  | Vegetables:  Napa cabbage, lettuce, Chinese cabbage, spinach, Chinese broccoli, baby Chinese cabbage, Chinese yam, taro, white radish, carrot, bean sprouts, tomato, winter melon, pumpkin, gourd, cucumber, and potato. |
|  | Proteins:  Lean pork, beef, lean lamb, chicken breast, and duck breast. |
|  | Fish and Seafood:  Yellow croaker, carp, black bass, grass carp, sea bass, pomfret, ribbonfish, mandarin fish, Chinese white shrimp, river shrimp, marsh shrimp, prawn, tiger shrimp, and white leg shrimp. |
|  | Fruits:  Apple, pear, peach, yellow peach, grape, orange, tangerine, pomelo, banana, watermelon, cantaloupe, kiwi, strawberry, and blueberry. |
|  | Oils:  Peanut oil, soybean oil, corn oil, canola oil, olive oil, sesame oil, and walnut oil. |
|  | Flavorings and Seasonings:  Green onion, ginger, garlic, lemon, cilantro, mint, salt, vinegar, star anise, fennel, bay leaves, and cinnamon. |
|  | Other Foods:  Chicken egg, duck egg, quail egg, tofu, soy milk. |
|  | **Disallowed Foods** |
|  | Frozen dumplings, frozen steamed buns, frozen wontons, instant porridge, instant rice, sour and spicy noodles, and instant rice noodles. |
|  | Frozen and canned vegetables and fruits. |
|  | Canned meat, ham, bacon, bloated fish, other parts of the chicken, pork, beef, lamb, duck (unless permitted above) |
|  | Dried fruit and candied fruit. |
|  | Alcoholic beverages, soft drinks, non-fresh fruit, vegetable juice, and dairy drink. |
|  | Animal fats, refined sugar, pickled foods, foods containing emulsifiers, artificial sweeteners, carrageenan, and xanthan gum. |
|  | Foods cooked by frying, deep-frying, or roasting. |
|  | **Restricted Foods** |
|  | Almonds, pine nuts, walnuts, peanuts, sunflower seeds, pistachios, hazelnuts, and macadamia nuts (based on gastrointestinal tolerance). |
| **Cooking Methods and Food Texture** | The First Week  Prepare a liquid diet using methods such as stewing, steaming, boiling, simmering, or braising. |
|  | The Second Week  Prepare semi-solid foods utilizing stewing, steaming, boiling, simmering, braising, stewing, and stir-frying. |
|  | The Third Week and Beyond  Prepare soft or regular food using methods such as stewing, steaming, boiling, simmering, braising, stir-frying, stir-frying in a thick sauce, blanching, and braising in sauce. |
